# Clinical AI Liability Literacy, Disclosure Confidence, and Adoption Readiness Among Romanian Healthcare Professionals: An Exploratory Single-Center Cross-Sectional Study

**DOI:** 10.3390/ejihpe16100153

**Published:** 2026-09-27

**Authors:** Daian-Ionel Popa, Ciprian Gîndac, Florina Buleu, Rotaru Roxana Elena, Codrina Mihaela Levai

**Affiliations:** 1 Research Center for Medical Communication, “Victor Babes” University of Medicine and Pharmacy, 300041 Timisoara, Romania; daian-ionel.popa@umft.ro (D.-I.P.);; 2 Municipal Emergency Hospital Timisoara, Eftimie Murgu Square No. 2, 300041 Timisoara, Romania; 3 Discipline of Medical Communications, Department II Microscopic Morphology, “Victor Babes” University of Medicine and Pharmacy, Eftimie Murgu Square No. 2, 300041 Timisoara, Romania; 4 Anesthesiology and Intensive Care Department, “Victor Babes” University of Medicine and Pharmacy, 300041 Timisoara, Romania; 5 Department of Cardiology, “Victor Babes” University of Medicine and Pharmacy, Eftimie Murgu Square No. 2, 300041 Timisoara, Romania; 6 New Line Medical Clinic Brasov, 500009 Brasov, Romania

**Keywords:** artificial intelligence, liability literacy, disclosure confidence, incident reporting, trust, adoption intention, health professions education, AI governance, exploratory study, cross-sectional survey

## Abstract

Background and Objectives: Errors generated by clinical artificial intelligence (AI) can obscure responsibility and complicate disclosure and incident reporting. This exploratory study examined AI-error liability literacy and its behavioral correlates among Romanian healthcare professionals. Materials and Methods: An exploratory, single-center cross-sectional survey was conducted from February to May 2026 among 109 clinical, managerial, legal/compliance, and IT/data professionals. No a priori sample-size calculation was performed; only moderate or larger effects were detectable. Participants completed a 20-item AI Error Liability Literacy Index and five-point behavioral measures. Analyses included Spearman correlations, k-means clustering, logistic regression, and covariate-adjusted mediation, with Firth penalization, role-adjusted, use-stratified, and competing-model sensitivity analyses, and bootstrap validation. Results: Mean liability literacy was 11.6 ± 4.2/20. Familiarity was higher for the General Data Protection Regulation (GDPR) (67.9%) than for the EU AI Act (24.8%). Literacy correlated with disclosure confidence (ρ = 0.49; 95% CI [0.33, 0.62]), an association that was attenuated but persisted after adjustment for professional role (partial ρ = 0.41), which correlated with adoption intention (ρ = 0.38), as did trust (ρ = 0.43); defensive stance correlated negatively (ρ = −0.31). Three exploratory profiles were identified (bootstrap Jaccard 0.71–0.82) that also differed on variables not used in clustering, and an indirect statistical association between literacy and adoption intention through disclosure confidence was observed (indirect effect 0.12; 95% CI [0.04, 0.22]), although concurrent measurement precludes causal or temporal interpretation. Under penalization, associations for literacy, trust, and disclosure confidence persisted; defensive stance and prior training did not. Conclusions: In this exploratory sample, adoption readiness was associated with regulatory knowledge, disclosure confidence, and calibrated trust. These hypothesis-generating findings require confirmation in larger, longitudinal studies before informing practice, and the appropriate goal is calibrated rather than maximal adoption.

## 1. Introduction

Artificial intelligence (AI) is increasingly embedded in clinical triage, diagnostic imaging, documentation, risk prediction, and surveillance for deterioration. Although these tools can augment professional judgment, false-positive or false-negative outputs, miscalibrated estimates, and misleading generated content may expose patients and organizations to harm. The consequences are not solely technical: an AI-related error raises behavioral questions about whether clinicians trust the system, feel able to challenge its output, and know how to communicate and report the incident. Human-centered implementation therefore requires both dependable technology and professionals who understand the boundaries of automated support (Topol, 2019; Wiens et al., 2019). Within the European Union, Regulation (EU) 2024/1689 (the AI Act) applies a risk-based framework to AI systems. Medical AI that is a safety component of, or itself constitutes, a regulated medical device requiring third-party conformity assessment may be classified as high-risk and become subject to risk management, human oversight, documentation, accuracy, robustness, and post-market monitoring requirements (European Parliament & Council of the European Union, 2017, 2024b). Processing health information also remains governed by the General Data Protection Regulation (GDPR) (European Parliament & Council of the European Union, 2016).

Despite clearer product and system obligations, the allocation of responsibility after AI-assisted harm remains difficult. Conventional negligence analysis generally assumes an identifiable human decision-maker, while clinical AI introduces opacity, probabilistic recommendations, evolving models, and shared control among clinicians, institutions, developers, and vendors. The European Commission’s 2022 proposal for an AI Liability Directive sought to address evidentiary barriers in non-contractual claims alongside the revised Product Liability Directive, but the proposal was withdrawn in 2025 (European Commission, 2022, 2025; European Parliament & Council of the European Union, 2024a). In practice, liability continues to be assessed through national rules and sector-specific regulation, leaving professionals uncertain about how much reliance is reasonable and who bears responsibility when an output contributes to harm (Maliha et al., 2021; Price et al., 2019). This uncertainty can encourage defensive behavior, delay adoption, or promote uncritical reliance if institutional roles are poorly defined. These attribution difficulties are likely to intensify as clinical AI moves beyond a single model producing a single recommendation toward agentic architectures in which multiple large language model (LLM) agents divide tasks, exchange intermediate outputs, retain memory across interactions, and jointly generate a decision (Hang et al., 2026; Sultimov et al., 2026). In such systems, tracing which agent, prompt, or handoff contributed to an erroneous output, and determining where human oversight should have intervened, becomes considerably more complex than for a conventional prediction model.

Acceptance of clinical AI is shaped by more than measured accuracy. The Technology Acceptance Model emphasizes perceived usefulness and usability, whereas the Consolidated Framework for Implementation Research and the NASSS framework place adoption within a broader system of individual beliefs, workflow compatibility, organizational capacity, and technological complexity (Damschroder et al., 2022; Greenhalgh et al., 2017; Holden & Karsh, 2010). Reviews and clinician surveys similarly identify trust, training, explainability, governance readiness, and medico-legal uncertainty as recurrent determinants of implementation (Castagno & Khalifa, 2020; Kelly et al., 2019; Lambert et al., 2023; Scheetz et al., 2021; Scott et al., 2021; Shaw et al., 2019). These determinants are behaviorally connected: limited knowledge may reduce confidence, perceived personal exposure may heighten defensive practice, and poorly calibrated trust may produce either rejection or overreliance (Asan et al., 2020; Longoni et al., 2019).

Confidence to disclose and report AI-related errors may be especially consequential but has received less empirical attention than general technology acceptance. Disclosure can be challenging when the basis for an output is difficult to explain, clinical and technical responsibilities are distributed, or the appropriate escalation pathway is unclear. Evidence from patient-safety research indicates that transparent communication, structured reporting, and a non-punitive learning environment are central to responding to medical errors, yet clinicians often remain uncertain about how and when to disclose them (Gallagher et al., 2003, 2007; Kaldjian et al., 2008). AI adds the need to reconstruct data provenance, system behavior, human oversight, and vendor responsibilities. Professionals who understand the applicable rules and know how to document and communicate an incident may therefore be more willing to use AI responsibly, whereas uncertainty may foster silence, blame, or avoidance.

We use the term AI Error Liability Literacy to denote a professional’s objective, action-oriented knowledge of how responsibility, evidence, and reporting duties are allocated when a clinical AI output contributes to harm. The construct is deliberately narrower than general regulatory knowledge, because it concentrates on the provisions that become operative after an error (human-oversight duties, record-keeping and traceability, serious-incident reporting, product and non-contractual liability, and the data-protection rules that govern automated decisions). It is also distinct from AI literacy as usually defined, which concerns understanding how AI systems work and how to use their outputs rather than who is answerable when they fail (Long & Magerko, 2020), and which the AI Act now requires providers and deployers to promote among their staff (European Parliament & Council of the European Union, 2024b). It differs, further, from prior training, which is an exposure that may or may not produce the knowledge, and from organizational preparedness, which is a property of the institution (for example, the availability of reporting pathways, measured separately here as reporting readiness) that may exist without individuals knowing how to use it. Liability literacy is thus a measurable attribute of the individual that can be assessed with right-or-wrong items. Positioning the construct in this way allows it to be examined as a candidate link between the governance environment and individual behavior, and thereby connects the technology-acceptance and implementation-science studies, which have concentrated on usefulness, usability, and organizational readiness, with the patient-safety literature on disclosure and reporting, in which knowledge of procedures is an established determinant of behavior.

Romania provides a useful setting for examining these relationships because public hospitals, private clinics, and academic centers vary in access to digital-health training, compliance expertise, and medico-legal support. Recent work in Romanian public healthcare has documented uneven awareness and implementation of GDPR requirements (Nastasa et al., 2025), but the behavioral implications of AI-specific liability knowledge have not been systematically evaluated. This study therefore aimed to (i) quantify AI-error liability literacy and compare it across professional roles; (ii) examine relationships among literacy, disclosure confidence, defensive stance, incident-reporting readiness, trust, and adoption intention; and (iii) identify implementation profiles with distinct educational and governance needs. We also explored whether disclosure confidence statistically accounted for part of the association between liability literacy and adoption intention. Because the study was conducted within a single institutional network with a modest convenience sample, it was designed, analyzed, and reported as an exploratory, hypothesis-generating investigation: the objectives were framed as estimation and description rather than as confirmatory testing, and no hypotheses were prespecified for inferential adjudication. The analysis was intended to generate candidate competency targets, which require confirmation in larger and more representative samples, for undergraduate, postgraduate, and continuing professional development programs in accountable clinical AI use.

## 2. Materials and Methods

### 2.1. Study Design and Setting

We performed a single-center, cross-sectional, questionnaire-based study from February through May 2026 among healthcare professionals affiliated with the “Victor Babeș” University of Medicine and Pharmacy, Timișoara, and its associated clinical network. Recruitment covered university-affiliated public hospital units, private clinics, and academic departments. Because decisions about AI procurement, deployment, use, incident review, and regulatory compliance span multiple functions, eligibility extended beyond front-line clinicians to managers, legal/compliance professionals, and IT/data personnel. The study is reported in accordance with the STROBE (Strengthening the Reporting of Observational Studies in Epidemiology) recommendations for cross-sectional studies (von Elm et al., 2007).

The protocol was approved by the Ethics Committee of the “Victor Babeș” University of Medicine and Pharmacy, Timișoara (No. 45, 26 January 2026), and the study was conducted in accordance with the Declaration of Helsinki. No personal identifiers were collected or retained. Before accessing the questionnaire, participants reviewed an electronic information and consent page. Participation was voluntary, anonymous, and uncompensated, and respondents could discontinue before submission without penalty.

### 2.2. Participants and Sampling

Adults aged ≥ 18 years employed in clinical care, healthcare management, legal/compliance, or health-IT roles within the participating network were eligible. Convenience recruitment was combined with role quotas to include both patient-facing and governance-facing perspectives. Respondents were excluded if consent was not provided or if missing responses prevented calculation of the primary outcomes. Among 118 individuals who opened the questionnaire, 109 (92.4%) contributed analyzable data; four declined consent, and five had more than 30% missing responses across the scored scales.

All eligible respondents with complete primary-outcome data were included in the analytical sample. An a priori sample-size calculation was not undertaken because the survey was intended as an exploratory description of one institutional network. The achieved sample was instead evaluated against the precision and power requirements of each planned analysis. A sensitivity power analysis (α = 0.05, two-sided; power = 0.80) indicated that *N* = 109 could detect Spearman correlations of |ρ| ≥ 0.27, standardized mean differences of d ≥ 0.55 for the comparison of participants with and without prior AI-error exposure (*n* = 43 vs. *n* = 66), and an omnibus effect of Cohen f ≥ 0.35 across the six professional roles; pairwise contrasts involving the smallest strata (e.g., legal/compliance, *n* = 11, vs. nurses, *n* = 27) required d ≥ 1.03. Because these minimum detectable effects were computed from the realized sample rather than from a prospective target, this “sensitivity” analysis is a post hoc (observed-power) description of the study’s precision limits and not an a priori power analysis; it was used only to frame interpretation. For the logistic model, 53 of 109 participants (48.6%) reported high adoption intention, yielding 4.1 events per estimated parameter in the fully adjusted specification (13 parameters) and 10.6 in a parsimonious specification (5 parameters); the former lies below the conventional minimum of 10 events per variable, and established criteria for the minimum sample size of binary-outcome models would require approximately 390 and 150 participants, respectively (Peduzzi et al., 1996; Riley et al., 2020). For the cluster analysis, the sample exceeded the rule-of-thumb minimum of two raised to the power of the number of clustering variables (64 for six variables) but not the more demanding recommendation of five times that value (320) (Formann, 1984). These benchmarks were used to frame interpretation rather than to justify the sample size, and all estimates involving smaller strata, the multivariable model, and data-derived profiles should be read as correspondingly imprecise. Accordingly, estimates for smaller professional strata and data-derived profiles were interpreted as hypothesis-generating rather than definitive.

### 2.3. Measures

The primary measure was a 20-item AI Error Liability Literacy Index (range 0–20), developed by mapping items to the EU AI Act, the 2022 AI Liability Directive proposal, the Product Liability Directive, the Medical Device Regulation, the GDPR, and Romanian Law no. 190/2018. These six instruments were combined because, taken together, they define the chain of obligations that becomes relevant once a clinical AI output contributes to harm: the AI Act specifies risk classification, human-oversight, logging, and serious-incident duties for providers and deployers; the Medical Device Regulation governs conformity, vigilance, and post-market surveillance for AI that qualifies as a medical device; the Product Liability Directive and the (since withdrawn) AI Liability Directive proposal address who compensates for defective or faulty AI and how evidence is obtained; and the GDPR, together with its Romanian implementing law, governs automated decision-making, health-data processing, and breach notification. Items were drafted to cover five content domains (AI Act obligations, 6 items; medical device regulation, 3 items; product and non-contractual liability, 4 items; data protection, 4 items; and cross-cutting disclosure, documentation, and escalation duties, 3 items). A mapping of each item to its content domain and primary legal source is provided in Appendix A (Table A1). Four academics with expertise in medical law, digital health, and clinical AI reviewed the content, and 11 healthcare professionals completed pilot testing to improve clarity and wording (European Commission, 2022; European Parliament & Council of the European Union, 2016, 2017, 2024a, 2024b). Each response was scored as correct or incorrect and summed, with higher totals representing greater objective literacy. Item-level content validity indices ranged from 0.82 to 1.00, the scale-level average content validity index was 0.93, and Kuder–Richardson 20 reliability was 0.77. Because random measurement error in a predictor attenuates rather than inflates its observed associations with other variables, a reliability of 0.77 implies that the correlations and regression coefficients reported for the index are, if anything, conservative estimates.

Five behavioral constructs were assessed on five-point Likert-type scales (1 = lowest; 5 = highest). Disclosure confidence captured perceived ability to explain, document, and report an AI-related error to patients and the institution. Defensive stance measured the inclination to alter practice primarily to limit personal medico-legal exposure. Incident-reporting readiness reflected perceived access to reporting pathways, templates, and oversight. Trust in AI represented beliefs about system reliability and institutional stewardship, whereas adoption intention measured near-term willingness to use or support AI despite the possibility of error. Adoption-intention items referred, for respondents not yet using AI, to willingness to begin using or to support the introduction of AI tools and, for current users, to willingness to continue or expand use and to support institutional deployment; current AI use was recorded separately (Table 1) so that its influence on the associations could be examined (Section 3.11). Trust, defensive stance, and reporting readiness were each represented by four-item mean scores, with Cronbach’s α values of 0.80, 0.78, and 0.81, respectively. Reverse-coded items were recoded so that higher values consistently indicated more of the named construct. The questionnaire preamble defined clinical AI broadly as software that generates predictions, classifications, recommendations, or text to support clinical or administrative decisions, giving diagnostic-imaging, risk-prediction, documentation, and generative (large language model) tools as examples; respondents were not asked to rate these categories separately.

### 2.4. Statistical Analysis

Continuous variables were described as mean ± standard deviation (SD) or median [interquartile range] when distributions were skewed; categorical variables were summarized as *n* (%). Normally distributed outcomes were compared using independent-samples t tests or one-way analysis of variance, and non-normal outcomes using Mann–Whitney U or Kruskal–Wallis tests. Because several attitudinal measures were ordinal and non-normally distributed, associations among continuous constructs were evaluated with Spearman rank correlations (ρ). Correlation coefficients are reported with 95% confidence intervals obtained by the Fisher z transformation so that the precision of each estimate is explicit. Because professional role differed on several measures, the pooled correlations were re-estimated in two role-adjusted sensitivity analyses: (i) partial Spearman correlations between the residuals of each measure after regression on professional-role indicator variables, and (ii) within-role correlations obtained from a bivariate random-intercept model with role as the grouping factor, which estimates the individual-level association net of between-role differences in means. Confidence intervals for the adjusted coefficients used the Fisher z transformation with degrees of freedom reduced by the number of role indicators. Shared method variance among the self-reported behavioral measures was screened with Harman’s single-factor test (Podsakoff et al., 2003).

Implementation profiles were generated by k-means clustering of z-standardized measures. Candidate solutions containing two through five clusters were compared using average silhouette width and substantive interpretability. Solutions were additionally compared using the gap statistic (500 reference datasets) and the Calinski–Harabasz index, and k-means was initialized with 1000 random starts so that the reported partition did not depend on a single local optimum (Rousseeuw, 1987; Tibshirani et al., 2001). Because the clustering variables are bounded Likert-type scores and a 0–20 count, all were z-standardized before clustering so that no measure dominated the Euclidean distance, and the k-means partition was compared with partitioning around medoids, which uses a medoid-based criterion that is less sensitive to the scale properties of ordinal inputs. Between-profile differences on the six clustering variables are expected by construction and are reported descriptively; as an external check, the profiles were additionally compared on variables that were not used in clustering (age, sex, sector, professional role, prior AI-error training, having witnessed an AI error, current AI use, and self-reported familiarity with the EU AI Act) using chi-square tests and one-way ANOVA. High adoption intention (score ≥ 4) was modeled with multivariable logistic regression that included prespecified covariates (age, sex, sector, and professional role) and the key predictors of liability literacy, trust, disclosure confidence, defensive stance, and prior AI-error training. Results are reported as adjusted odds ratios (aORs) with 95% confidence intervals (CIs). Because the number of outcome events was small relative to the number of estimated parameters, three robustness analyses were added: (i) Firth penalized (bias-reduced) maximum-likelihood estimation of the same fully adjusted model; (ii) a parsimonious model restricted to the five key predictors without demographic covariates; and (iii) internal validation by bootstrap resampling (1000 replications) to obtain optimism-corrected discrimination and a calibration slope (Firth, 1993; Heinze & Schemper, 2002; Steyerberg, 2019). Multicollinearity was assessed with variance inflation factors, calibration with the Hosmer–Lemeshow test, and the stability of predictor selection by recording how often each variable was retained by backward elimination across 1000 bootstrap samples. A covariate-adjusted mediation model evaluated the statistical pathway from literacy to adoption intention through disclosure confidence, with 5000 bootstrap resamples used to estimate the bias-corrected indirect effect. The mediation estimate was further examined with a sensitivity analysis for unmeasured mediator–outcome confounding, expressed as the residual correlation at which the indirect effect would no longer differ from zero, and its power was evaluated against published requirements for detecting mediated effects (Fritz & MacKinnon, 2007; Imai et al., 2010). Because all variables were measured concurrently, the mediation model is a decomposition of association rather than a test of a causal or temporal pathway; to make this dependence on assumed ordering explicit, two competing specifications were also estimated in which the roles of literacy and disclosure confidence were exchanged and in which adoption intention was treated as the intermediate variable. Finally, the principal analyses were repeated after stratifying by, and adjusting for, current AI use in the respondent’s workflow. All tests were two-sided, and *p* < 0.05 was considered statistically significant. All analyses were exploratory, and no adjustment for multiple comparisons was applied; *p*-values are therefore interpreted descriptively, alongside effect sizes and confidence intervals, rather than as formal tests of prespecified hypotheses. Missing data were not imputed beyond the prespecified exclusion rule. Cluster stability was evaluated by non-parametric bootstrap resampling (1000 replications), summarized as the mean Jaccard similarity of each profile and the proportion of replications in which a profile dissolved (Jaccard < 0.50), and by comparing the k-means partition with Ward hierarchical and partitioning-around-medoids solutions using the adjusted Rand index; a leave-one-variable-out procedure assessed dependence on any single clustering measure (Hennig, 2007; Hubert & Arabie, 1985). Analyses were performed in R version 4.3.1 (R Foundation for Statistical Computing, Vienna, Austria) with the packages logistf (version 1.26.0), rms (version 6.7-1), cluster (version 2.1.4), fpc (version 2.2-10), and mediation (version 4.5.0).

## 3. Results

### 3.1. Participant Characteristics

The final sample included 109 participants, with a mean age of 39.4 ± 10.1 years; 67 (61.5%) were women (Table 1). Just over half worked in public hospital settings (51.4%), while 28.4% were based in private clinics and 20.2% in academic settings. Physicians (30.3%), nurses (24.8%), and allied health professionals (11.0%) represented the principal clinical groups; administrators/managers, legal/compliance staff, and IT/data personnel supplied organizational and technical perspectives. Previous AI-error training was reported by 35.8%, an AI error or near-miss had been witnessed by 39.4%, and 43.1% reported using AI in their workflow.

### 3.2. Outcomes by Professional Role

Professional groups differed in liability literacy, disclosure confidence, and reporting readiness (Table 2; Figure 1). Legal/compliance respondents had the highest mean literacy (16.1 ± 2.4), disclosure confidence (3.8 ± 0.6), and reporting readiness (3.6 ± 0.6). IT/data personnel had the highest adoption-intention score (3.9 ± 0.6), whereas nurses had the lowest mean literacy (9.8 ± 3.6), disclosure confidence (2.8 ± 0.8), and reporting readiness (2.6 ± 0.7). Adoption intention itself did not vary significantly by professional role (*p* = 0.302). Expressed as η^2^, professional role accounted for 22% of the variance in liability literacy, 13% in disclosure confidence, and 16% in reporting readiness, but only 6% in adoption intention. Because several role strata were small, these means are imprecise: the 95% confidence interval for liability literacy spanned 14.5–17.7 among legal/compliance staff (*n* = 11) and 11.6–15.8 among IT/data personnel (*n* = 10), compared with 11.3–13.9 among physicians (*n* = 33). Role-level comparisons are therefore descriptive.

### 3.3. Familiarity with Regulatory Frameworks

Awareness was most frequently reported for the GDPR (67.9%), followed by the Medical Device Regulation (45.0%) and Romanian Law no. 190/2018 (30.3%) (Table 3). Only 24.8% indicated familiarity with the EU AI Act, and 20.2% with the 2022 AI Liability Directive proposal. Thus, fewer than one quarter of respondents reported awareness of either AI-specific instrument. These self-ratings should not be interpreted as equivalent to performance on the objective liability-literacy index.

### 3.4. Outcomes by Prior AI-Error Experience

Respondents who had witnessed an AI error or near-miss scored higher on liability literacy (13.1 ± 3.6 vs. 10.7 ± 4.1; *p* = 0.002), disclosure confidence (3.4 ± 0.8 vs. 2.9 ± 0.8; *p* = 0.002), reporting readiness (3.2 ± 0.7 vs. 2.8 ± 0.8; *p* = 0.007), and defensive stance (3.6 ± 0.7 vs. 3.2 ± 0.8; *p* = 0.007) (Table 4). Adoption intention was similar between groups (*p* = 0.505), indicating that prior error exposure coincided with greater preparedness and caution but not lower willingness to adopt AI.

### 3.5. Correlation Structure

Liability literacy was positively associated with disclosure confidence (ρ = 0.49; 95% CI [0.33, 0.62]; *p* < 0.001) and reporting readiness (ρ = 0.34; 95% CI [0.16, 0.50]; *p* < 0.001) (Table 5; Figure 2). Adoption intention correlated positively with disclosure confidence (ρ = 0.38; *p* < 0.001) and trust in AI (ρ = 0.43; 95% CI [0.26, 0.57]; *p* < 0.001), and negatively with defensive stance (ρ = −0.31; 95% CI [−0.47, −0.13]; *p* = 0.001). Defensive stance was also inversely related to disclosure confidence (ρ = −0.27; 95% CI [−0.44, −0.09]; *p* = 0.005). These concurrent associations describe covariance among the constructs and do not establish directionality. Because these coefficients pool respondents across six professional roles that differ in mean literacy, confidence, and readiness (Table 2), part of each pooled coefficient could reflect between-role composition rather than individual-level covariation. Table 6 therefore reports role-adjusted estimates. Adjustment for role attenuated the literacy–disclosure confidence association from ρ = 0.49 to a partial ρ of 0.41 (95% CI [0.24, 0.56]) and a within-role (random-intercept) ρ of 0.40 (95% CI [0.22, 0.55]), and the literacy–reporting readiness association from 0.34 to 0.27 (95% CI [0.08, 0.44]); the associations involving adoption intention, which itself did not differ by role, changed by only 0.02–0.03. Approximately one sixth of the pooled literacy–confidence coefficient was therefore attributable to between-role differences, but the majority reflected covariation among individuals within the same role. Harman’s single-factor test on the behavioral items indicated that the first unrotated factor accounted for 31.4% of the total variance, below the conventional 50% threshold, although this test is insensitive and cannot exclude shared method variance (Section 4.4). The intervals are wide and, for the weaker associations, approach the null value, so the magnitude of these relationships should be regarded as imprecisely estimated.

### 3.6. Predictors of High Adoption Intention

In the adjusted logistic model, high adoption intention was associated with greater liability literacy (aOR = 1.78 per 1-SD increase; 95% CI [1.16, 2.73]; *p* = 0.008), higher trust (aOR = 1.69; 95% CI [1.10, 2.59]; *p* = 0.016), stronger disclosure confidence (aOR = 1.61; 95% CI [1.04, 2.49]; *p* = 0.033), a lower defensive stance (aOR = 0.66; 95% CI [0.44, 0.98]; *p* = 0.042), and previous AI-error training (aOR = 2.04; 95% CI [1.01, 4.12]; *p* = 0.047) (Table 7). Age, sex, and public-sector employment were not significantly related to high adoption intention, suggesting that modifiable knowledge and attitudinal factors distinguished likely adopters more clearly than the measured demographic characteristics. These estimates should, however, be read with the precision of the model in mind: 13 parameters were estimated from 53 events (4.1 events per variable), and the intervals for prior AI-error training (1.01–4.12) and defensive stance (0.44–0.98) lie close to unity. Sensitivity analyses addressing this limitation are reported in Section 3.9.

### 3.7. Implementation Profiles

K-means analysis produced three implementation profiles (Table 8; Figure 3). Because the six measures in Table 8 were themselves the clustering inputs, the fact that the profiles differ on them (all one-way ANOVA *p* < 0.001) is expected by construction and is not independent evidence that the profiles are substantively distinct; the informative content of the solution lies in the configuration of scores within each profile rather than in the between-profile *p*-values. Accountable Adopters (*n* = 41; 37.6%) combined the highest literacy, disclosure confidence, reporting readiness, trust, and adoption intention with the lowest defensive stance. Defensive Compliers (*n* = 39; 35.8%) had intermediate literacy and adoption scores but the highest defensive stance. The distinctive feature of this profile is thus a combination (moderate literacy and adoption alongside the strongest self-protective orientation) rather than a uniformly low or high position on all measures. Unprepared Skeptics (*n* = 29; 26.6%) showed the lowest literacy, confidence, readiness, trust, and adoption intention. Given the modest single-center sample, these profiles should be viewed as exploratory; their internal stability was formally assessed by resampling and is reported in Section 3.10, together with an external comparison on variables that were not used to construct the profiles.

### 3.8. Mediation Analysis

In the covariate-adjusted mediation model, an indirect statistical association between liability literacy and adoption intention through disclosure confidence was observed (Table 9). The path from literacy to disclosure confidence was significant (β = 0.43; SE = 0.08; *p* < 0.001), as was the path from disclosure confidence to adoption intention (β = 0.27; SE = 0.09; *p* = 0.003). Literacy retained an adjusted direct association with adoption intention (c′: β = 0.16; SE = 0.07; *p* = 0.024), and the bootstrapped indirect effect was 0.12 (95% CI [0.04, 0.22]). Because exposure, mediator, and outcome were measured simultaneously, these estimates represent a decomposition of association rather than a causal or temporal sequence. The estimate is also limited in precision: a sample of 109 provides roughly 70% power to detect an indirect effect of this magnitude when the a path is moderate and the b path small-to-moderate, and the indirect effect ceased to differ from zero once the residual correlation between mediator and outcome errors reached 0.21, indicating sensitivity to modest unmeasured confounding. To show how far the interpretation depends on the assumed ordering, two competing specifications were estimated with the same covariates (Table 10). Exchanging the roles of literacy and disclosure confidence (confidence → literacy → adoption) produced an indirect estimate of 0.07 (95% CI [0.01, 0.15]), and treating adoption intention as the intermediate variable (literacy → adoption → confidence) produced an indirect estimate of 0.07 (95% CI [0.01, 0.14]). Because the three specifications are statistically equivalent given cross-sectional data, and each yielded a non-zero indirect estimate, the data cannot discriminate among them; the literacy → confidence → adoption ordering is retained as the theoretically motivated specification, but the results should be read as an indirect statistical association rather than as evidence of a mediating pathway.

### 3.9. Precision and Sensitivity of the Multivariable Model

Because the fully adjusted model estimated 13 parameters from 53 events, three robustness analyses were performed (Table 11). Firth penalized estimation produced slightly attenuated but directionally identical coefficients, and the associations for liability literacy (aOR = 1.69; 95% CI [1.12, 2.58]), trust (aOR = 1.62; 95% CI [1.07, 2.45]), and disclosure confidence (aOR = 1.54; 95% CI [1.02, 2.34]) remained statistically significant. By contrast, the associations for defensive stance (aOR = 0.70; 95% CI [0.48, 1.02]) and prior AI-error training (aOR = 1.88; 95% CI [0.95, 3.72]) crossed unity under penalization, indicating that these two findings are not robust to the small number of events. The parsimonious model, which achieved 10.6 events per variable, reproduced the pattern of the fully adjusted model, suggesting that the principal associations were not artifacts of covariate over-adjustment. Multicollinearity was low (maximum variance inflation factor = 1.74) and calibration was acceptable (Hosmer–Lemeshow *p* = 0.42), but internal validation revealed appreciable optimism: apparent discrimination of 0.79 fell to 0.72 after bootstrap correction, with a calibration slope of 0.78, consistent with the overfitting expected at this sample size. Across 1000 bootstrap samples, backward elimination retained liability literacy in 84.6% and trust in 79.2% of replications, whereas disclosure confidence (66.1%), defensive stance (58.3%), and prior AI-error training (51.7%) were retained less consistently. A post hoc sensitivity (observed-power) analysis based on the realized sample indicated that the study could detect odds ratios of approximately 1.71 per standard deviation at 80% power, rising to about 1.90 once shared variance among predictors is taken into account; several observed estimates lie at or below this threshold and are therefore preliminary.

### 3.10. Robustness of the Cluster Solution

Internal validation identified the three-cluster solution as the best of the candidate partitions while showing that its separation is only moderate (Table 12). Average silhouette width was highest at k = 3 (0.41), and the gap statistic and the Calinski–Harabasz index selected the same optimum; the partition was reproduced in 96.4% of 1000 random initializations. Bootstrap resampling indicated that Accountable Adopters were stable (mean Jaccard = 0.82), Unprepared Skeptics were stable by the same criterion but close to its lower boundary (0.76; dissolved in 5.3% of replications), and Defensive Compliers were less certain (0.71), the latter dissolving in 8.4% of replications. Agreement with alternative algorithms was moderate to substantial (Ward hierarchical clustering: adjusted Rand index = 0.68, 84.4% identical assignments; partitioning around medoids: adjusted Rand index = 0.73, 87.2% identical assignments), and no single variable determined the solution in the leave-one-variable-out analysis. These procedures test whether the partition is reproducible; they do not test whether the profiles differ on anything other than their own inputs. Table 13 therefore compares the profiles on variables that were not used in clustering. The profiles did not differ in age, sex, sector, or clinical versus non-clinical role composition, but they did differ in prior AI-error training (51.2% of Accountable Adopters vs. 33.3% of Defensive Compliers and 17.2% of Unprepared Skeptics; *p* = 0.013), current AI use (61.0% vs. 38.5% vs. 24.1%; *p* = 0.007), self-reported familiarity with the EU AI Act (39.0% vs. 20.5% vs. 10.3%; *p* = 0.018), and having witnessed an AI error or near-miss (48.8% vs. 46.2% vs. 17.2%; *p* = 0.016). The last of these is informative about the Defensive Compliers profile: its members were as likely as Accountable Adopters to have witnessed an AI error, yet reported a markedly more self-protective orientation, which is consistent with the interpretation of this profile as a distinct behavioral configuration rather than an intermediate point on a single continuum. Effect sizes for these external differences were small to moderate (Cramér’s V 0.27–0.30); the comparisons were not prespecified, and they do not substitute for validation in an independent sample. However, with six clustering variables and a smallest profile of 29 participants, the analysis provided fewer than five observations per variable within that profile, below recommended thresholds. The profiles should therefore be treated as a provisional heuristic for organizing educational needs rather than as validated participant types, and the boundary between Defensive Compliers and the other two groups in particular requires confirmation in independent samples.

### 3.11. Sensitivity Analysis by Current AI Use

Because 47 respondents (43.1%) already used AI in their workflow, the principal analyses were repeated after distinguishing current users from non-users (Table 14). Current users reported higher adoption intention (3.9 ± 0.6 vs. 3.3 ± 0.8; *p* < 0.001; d = 0.83), liability literacy (12.8 ± 4.0 vs. 10.7 ± 4.1; *p* = 0.009), trust (3.5 ± 0.6 vs. 3.1 ± 0.7; *p* = 0.002), and reporting readiness (3.1 ± 0.7 vs. 2.8 ± 0.8; *p* = 0.040), with a smaller difference in disclosure confidence (3.3 ± 0.8 vs. 3.0 ± 0.9; *p* = 0.069) and none in defensive stance (*p* = 0.520). High adoption intention was reported by 30 of 47 users (63.8%) and 23 of 62 non-users (37.1%). Within each stratum, however, the correlations of adoption intention with liability literacy (users, ρ = 0.31; non-users, ρ = 0.33), disclosure confidence (0.35; 0.39), and trust (0.40; 0.44) were of similar magnitude and direction, although the stratum-specific intervals were wide. Adding current AI use to the fully adjusted logistic model left the estimates for literacy (aOR = 1.72; 95% CI [1.10, 2.69]), trust (aOR = 1.63; 95% CI [1.05, 2.53]), and disclosure confidence (aOR = 1.57; 95% CI [1.00, 2.46]) essentially unchanged, while current use itself was associated with high adoption intention (aOR = 2.61; 95% CI [1.12, 6.08]); interaction terms between current use and each key predictor were not significant (all *p* ≥ 0.41). The indirect estimate in the primary mediation model was 0.11 (95% CI [0.03, 0.21]) after additional adjustment for current use. Current use therefore appears to shift the level of adoption intention without materially altering its associations with literacy, confidence, and trust, although the cross-sectional design cannot determine whether use produced literacy and trust or the reverse.

## 4. Discussion

### 4.1. Principal Findings

This exploratory study describes associations between regulatory knowledge about AI-related harm and behavioral readiness to use clinical AI. Objective liability literacy was moderate, and familiarity with the GDPR substantially exceeded awareness of the EU AI Act. Literacy was related to both disclosure confidence and reporting readiness, while adoption intention was independently associated with literacy, trust, disclosure confidence, lower defensive stance, and prior AI-error training. In penalized sensitivity models, however, only the associations with literacy, trust, and disclosure confidence remained statistically significant. The bivariate associations were attenuated but not eliminated after adjustment for professional role (Table 6), and the estimates for literacy, trust, and confidence were essentially unchanged when current AI use was added to the model (Table 14), so neither between-role composition nor prior use appears to account for them. The results are therefore compatible with the possibility that accountable adoption is not simply a matter of favorable attitudes toward technology, but may also relate to whether professionals understand the applicable rules and feel capable of acting when an AI-supported decision goes wrong. Given the small single-center sample, the width of the confidence intervals, and the attenuation of several estimates under penalized regression, this interpretation remains provisional. It is further qualified by the fact that all behavioral constructs were self-reported in a single questionnaire, so part of their intercorrelation, and of the regression and mediation estimates built on it, may reflect shared method variance (Section 4.4).

The professional-role pattern is also of interest, although the role-specific estimates were imprecise. Legal/compliance personnel were the most knowledgeable and most confident about disclosure and reporting, whereas nurses recorded the lowest mean scores on these measures. However, adoption intention did not differ significantly across roles. One plausible explanation for this apparent tension (strong role effects on the predictors but not on the outcome) is that adoption intention is shaped by several influences that are distributed differently across roles: legal/compliance staff were the most literate and confident but reported the lowest adoption intention of any group (3.3 ± 0.7), whereas IT/data personnel reported the highest (3.9 ± 0.6), and role accounted for only 6% of the variance in adoption intention compared with 22% in literacy. Knowledge of liability rules may therefore raise intention among clinicians who see AI as a tool they will use while raising caution among those whose function is to anticipate institutional exposure, and role also captures differences in workflow relevance and trust that are not reducible to literacy. The within-role correlations (Table 6) confirm that the individual-level literacy–confidence association is present inside roles as well as between them. This combination raises the possibility that willingness may exist even where preparedness is limited, which could create an implementation risk if access to AI expands faster than education and governance support. Participants who had encountered an AI error or near-miss showed greater literacy, confidence, readiness, and defensiveness, but not lower adoption intention. Error exposure may therefore promote learning and vigilance without necessarily producing wholesale rejection of AI, a pattern consistent with concerns about unintended consequences and the human-factors demands of safe clinical implementation (Cabitza et al., 2017; Sujan et al., 2019).

The cluster solution translated these continuous relationships into three exploratory implementation profiles. Accountable Adopters appeared ready to use AI within an oversight framework; Defensive Compliers combined moderate preparedness with pronounced self-protective attitudes; and Unprepared Skeptics had low knowledge, trust, confidence, and readiness. Bootstrap validation indicated that the Accountable Adopters profile was the most stable and the Defensive Compliers profile the least, so this three-group structure should be read as provisional. Because the six variables that describe the profiles are the same variables from which they were constructed, the between-profile differences in Table 8 are expected by construction; the additional value of the solution lies in the configuration of each profile, particularly the pairing of a pronounced defensive stance with only moderate literacy among Defensive Compliers, and in the finding that the profiles also differed on training, current use, regulatory familiarity, and error exposure, none of which entered the algorithm (Table 13). Even so, the profiles remain a description of this sample rather than a stable typology and should not be read as establishing that such types exist beyond it. The mediation analysis further indicated an indirect statistical association between literacy and adoption intention through disclosure confidence, although this estimate was sensitive to modest unmeasured confounding, and competing specifications with the ordering reversed produced indirect estimates of similar magnitude (Table 10). Although the cross-sectional design precludes causal or temporal interpretation and the data cannot distinguish this ordering from its alternatives, the pattern is compatible with a practical mechanism in which knowledge becomes behaviorally relevant when professionals can convert it into documentation, communication, and reporting actions.

### 4.2. Interpretation in Relation to Prior Evidence

These findings extend technology-acceptance research by positioning liability literacy as a potentially modifiable implementation factor. Established models emphasize usefulness, usability, organizational readiness, and system complexity (Damschroder et al., 2022; Greenhalgh et al., 2017; Holden & Karsh, 2010), while empirical reviews show that clinician acceptance is shaped by knowledge, training, workflow fit, and institutional support (Castagno & Khalifa, 2020; Lambert et al., 2023; Scheetz et al., 2021). The present results add that professionals may be more willing to adopt AI when they understand the accountability environment and perceive that errors can be managed through established processes. This interpretation also aligns with work demonstrating resistance to medical AI when algorithmic recommendations appear to neglect individual clinical circumstances or diminish professional agency (Longoni et al., 2019). The specific contribution of the present construct is to isolate, and to measure objectively, the after-the-error component of the accountability environment: prior work has treated medico-legal uncertainty as a perceived barrier, whereas liability literacy is an assessable competency that institutions can teach and audit, and that can be tested as a potential link between governance arrangements and individual behavior.

The association of disclosure confidence with adoption intention connects AI implementation to the broader patient-safety literature. Effective disclosure requires timely recognition of harm, an understandable explanation, acknowledgment of uncertainty, and a plan for remediation. Studies of conventional medical errors have shown that patients generally expect transparent communication, while physicians may hesitate because of uncertainty, emotional consequences, or liability concerns (Gallagher et al., 2003, 2007). Reporting studies similarly show that knowledge of procedures and a supportive safety culture influence whether clinicians document incidents (Kaldjian et al., 2008). AI-related events add technical questions—such as data provenance, model version, output traceability, and the degree of human review—that can make disclosure and reporting more difficult unless institutions provide specific templates and multidisciplinary support.

Trust also remained independently associated with adoption intention, but trust should be calibrated rather than maximized. Excessive confidence can foster automation bias and overreliance, whereas opaque or clinically irrelevant explanations may reduce trust without improving safety (Amann et al., 2020; Ghassemi et al., 2021; Rosenbacke et al., 2024). Human-centered AI therefore requires systems that communicate uncertainty, preserve opportunities to question or override outputs, and make the basis and limitations of recommendations visible at the point of care (Asan et al., 2020; Topol, 2019). The negative association between defensive stance and adoption intention is consistent with this view: when professionals perceive AI primarily as a source of personal exposure, they may avoid beneficial tools even when technical performance is acceptable, echoing broader evidence on defensive medicine and liability-related behavior (Studdert et al., 2005).

A related point is that adoption intention should not be read as desirable in itself. The analyses in this study treat higher adoption intention as the outcome of interest because it is the behavioral end-point that governance and education are most often intended to influence, but willingness to use AI is beneficial only when the system is adequately validated, appropriate for the task, and used with a degree of reliance that matches its performance; willingness to adopt a poorly validated tool, or to rely on an appropriate tool excessively, is a safety hazard rather than a success. The more defensible target is therefore calibrated adoption, in which the intention to use AI is conditional on system performance, clinical context, human oversight, and the availability of escalation mechanisms, rather than maximal adoption. Seen in this light, the observation that adoption intention was associated with liability literacy, disclosure confidence, and trust, and that respondents who had witnessed an AI error were more cautious without being less willing to adopt, is encouraging precisely because it suggests that willingness in this sample tended to accompany, rather than substitute for, awareness of accountability. The study did not, however, measure whether respondents’ intentions were conditional on system characteristics, and future instruments should ask directly about the circumstances under which professionals would decline to use, override, or escalate an AI output.

The regulatory context may intensify these behavioral responses. Legal responsibility for an AI-assisted decision can be distributed across professionals, healthcare organizations, manufacturers, and software providers, and existing rules do not always map neatly onto adaptive or probabilistic systems (Maliha et al., 2021; Price et al., 2019). Product-safety and medical-device obligations remain essential, but they do not by themselves specify how a clinician should disclose an event, preserve evidence, or escalate a suspected model failure. The gap observed between GDPR familiarity and AI-specific awareness may reflect prior institutional emphasis on data protection rather than on the newer lifecycle obligations of the AI Act. Education should therefore distinguish privacy compliance from the wider domains of risk management, human oversight, post-market monitoring, and incident response.

### 4.3. Implications for Governance, Education, and Practice

For healthcare organizations, the results are consistent with, but cannot by themselves establish the effectiveness of, a governance model in which accountability is shared and operationalized before deployment. Each AI system should have a named clinical owner, documented indications and limitations, criteria for human override, an audit trail, and a clear route for escalating suspected errors. Procurement and implementation teams should define which events require internal reporting, regulatory notification, vendor review, or patient disclosure. These measures are consistent with system-level approaches to AI safety and governance that emphasize continuous monitoring rather than one-time validation (Gerke et al., 2020; Reddy et al., 2020; Shankar, 2026; World Health Organization, 2021, 2023). A closely related allocation of responsibility across clinicians, institutions, and developers, with named ownership, defined escalation routes, and role-specific competencies, has recently been proposed as a professional-accountability framework for AI-assisted nursing practice (Shankar, 2026); it addresses the same distributed-responsibility problem that the liability-literacy construct is designed to measure readiness for.

The findings suggest that education could usefully be role-specific but interprofessional. Clinicians need practical instruction in recognizing unsafe outputs, documenting the human decision process, communicating uncertainty, and initiating incident reports. Managers require competencies in workflow redesign and safety culture; legal/compliance staff in translating regulation into usable protocols; and IT/data teams in model monitoring, version control, and post-incident reconstruction. Simulation-based exercises using realistic false-negative, false-positive, and generative-AI scenarios may be more effective than purely didactic instruction because they require participants to decide who should be contacted, what should be documented, and how responsibility should be communicated. The role pattern observed here suggests that such education should be differentiated in content but shared in structure. Nurses, who reported the lowest literacy, confidence, and readiness despite an adoption intention comparable to that of other groups, may benefit most from practical, scenario-based training in recognizing and reporting AI-related incidents and in documenting the human decision process; legal/compliance staff, who were the most literate but the least inclined to adopt, may need a better understanding of clinical workflow, model limitations, and the practical consequences of over-cautious policy; and IT/data personnel, who were the most willing adopters, may need explicit grounding in disclosure and patient-communication duties. Interdisciplinary formats (joint incident reviews, simulation with mixed clinical, managerial, legal, and technical participants, and shared governance committees) would allow each group to supply the competency the others lack and would make the distribution of responsibility visible in practice rather than only in policy documents. Uniform, role-blind curricula risk under-serving the groups with the largest gaps while adding little for those already familiar with the regulatory material.

The durability of liability literacy as an educational competency also deserves comment, because the European regulatory landscape is still evolving: the AI Act’s obligations are being phased in, the Product Liability Directive has only recently been recast, and the AI Liability Directive proposal on which several index items drew was withdrawn in 2025. Knowledge of specific provisions will therefore date quickly. Educational programs should accordingly aim less at memorizing the content of individual instruments than at developing adaptive regulatory literacy: knowing where to locate the currently applicable requirements, understanding how responsibilities are allocated among the clinician, the institution, and the manufacturer, documenting human oversight in a way that will withstand later scrutiny, preserving the evidence needed to reconstruct an event, and recognizing when legal or compliance consultation is required. Several index items already target these procedural capabilities rather than provision-specific facts (Appendix A), and future versions of the instrument should shift further in this direction so that the competency it measures remains meaningful as the specific rules change.

Organizations could also consider designing non-punitive reporting processes that capture both patient harm and near-misses. A useful AI-event report would record the clinical context, input data, system version, output, clinician response, override status, downstream consequences, and any communication with the patient or vendor. Linking these reports to multidisciplinary review can transform individual uncertainty into organizational learning while reducing the “problem of many hands” in complex systems (Dixon-Woods & Pronovost, 2016; Habli et al., 2020). Such infrastructure may also reduce defensive behavior by making responsibility visible and distributed rather than implicitly assigning all risk to the final user.

This distributed-responsibility problem is likely to become more acute with the emergence of agentic and multi-agent LLM systems. Recent work outside healthcare shows that multiple LLM agents can be organized as teammates that divide a problem, exchange partial solutions, and jointly produce an answer (Hang et al., 2026), and that an LLM-based coordinator can direct many subordinate agents while drawing on episodic memory of previous situations and exposing its reasoning for human audit (Sultimov et al., 2026). If comparable architectures reach clinical settings (for example, an orchestrating agent that delegates retrieval, summarization, and recommendation to specialized agents and retains memory across encounters), the questions that liability literacy addresses (who was responsible for oversight, which intermediate step introduced the error, what was logged, and when a human should have intervened) will multiply across agents, handoffs, and memory states. Governance frameworks, and the competencies they require of staff, will need to account for delegation, inter-agent handoffs, traceability of intermediate reasoning, and accountability that is distributed across agents as well as across people, rather than assuming that clinical AI is a single isolated decision-support model. The present instrument was developed for the latter case and would need extension to capture these additional dimensions.

The exploratory profiles provide one way to tailor implementation. Accountable Adopters may benefit from advanced scenario testing and participation in governance committees. Defensive Compliers may need explicit role allocation, psychological safety, and reassurance that appropriate reporting will not automatically be treated as individual fault. Unprepared Skeptics may require foundational legal and technical literacy, supervised exposure, and opportunities to build calibrated trust. These categories should not be used as fixed labels; rather, they can guide the intensity and sequencing of education while institutions evaluate whether individuals move between profiles after training or changes in governance. Because the profiles derive from a single exploratory sample and showed only moderate internal stability, they should inform local needs assessment rather than any formal triage or appraisal of staff. More broadly, the practical suggestions in this section are derived from cross-sectional associations in one network and should be regarded as hypotheses for implementation research rather than as evidence-based practice guidance.

### 4.4. Limitations and Future Research

Several limitations constrain interpretation. The single-center, convenience-sampled design limits generalizability and may have attracted respondents with greater interest in AI or regulation. The overall sample was modest, and the legal/compliance and IT/data groups were small, reducing precision for role comparisons. The post hoc sensitivity (observed-power) analysis showed that the study could detect only moderate or larger effects (|ρ| ≥ 0.27; d ≥ 0.55; odds ratios ≥ 1.71 per standard deviation), so smaller but potentially meaningful associations may have gone undetected, and contrasts involving the smallest role strata were powered only for very large differences (d ≥ 1.03). Sample size was determined by the number of eligible respondents in one network rather than by a prespecified target, and the study is consequently descriptive rather than confirmatory. All attitudes and prior experiences were self-reported and may have been influenced by recall, social desirability, or local organizational culture. Because disclosure confidence, reporting readiness, trust, defensive stance, and adoption intention were all collected in the same questionnaire at the same time, common-method variance and perceptual overlap among these constructs are a further concern: they may share response tendencies such as generalized optimism about AI or confidence in one’s institution, so the correlations among them (for example, between disclosure confidence and adoption intention, or between trust and adoption intention) and the corresponding regression and mediation estimates may partly reflect shared measurement context rather than fully distinct behavioral mechanisms (Podsakoff et al., 2003). Harman’s single-factor test did not indicate a dominant common factor (31.4% of variance), but this test has limited sensitivity, and the objective literacy index is the only measure not subject to this concern. Respondents may also have interpreted “clinical AI” differently: the term encompasses conventional prediction models, diagnostic imaging systems, documentation tools, generative and large language model applications, and semi-autonomous or agentic decision-support systems, whose error types, explainability requirements, and liability pathways differ considerably, and the questionnaire did not ask participants to distinguish among them. The reported associations therefore describe attitudes toward clinical AI in general and may not generalize equally to each technology class. The study measured adoption intention rather than observed AI use, disclosure behavior, or incident reporting. Although the associations of interest were similar among current users and non-users of AI (Table 14), the study cannot determine whether prior use generated literacy and trust or the reverse.

The liability-literacy index underwent expert review, pilot testing, and internal-consistency assessment, but further psychometric validation is required across institutions and professions. Its internal consistency (KR-20 = 0.77) is acceptable but modest; because random measurement error in a predictor attenuates rather than inflates its associations, this imprecision would tend to make the reported coefficients involving the index conservative (correcting ρ = 0.49 for unreliability of the index alone would yield approximately 0.56), but it also reduces the precision of the downstream models in which the index is used. The exploratory analyses included multiple comparisons without multiplicity adjustment, and the fully adjusted logistic model provided only 4.1 events per estimated parameter, below the conventional minimum of 10. Bootstrap internal validation confirmed appreciable optimism (c-statistic 0.79 apparent vs. 0.72 corrected; calibration slope 0.78), and the associations for defensive stance and prior AI-error training did not persist under Firth penalization, so those two findings in particular should be considered unconfirmed. K-means clustering can produce sample-dependent solutions. Euclidean k-means also treats bounded, ordinal Likert-type scores as continuous and equally spaced, which can distort distances near the scale limits; standardization and the substantial agreement with a medoid-based algorithm (adjusted Rand index = 0.73) mitigate but do not remove this concern, and latent profile analysis or distance measures designed for ordinal data would be preferable in larger samples. Although bootstrap resampling, alternative clustering algorithms, and leave-one-variable-out analyses supported the three-profile structure, the Defensive Compliers profile was only moderately stable; the smallest profile contained fewer than five observations per clustering variable; and, although the profiles differed on several variables that were not used in clustering (Table 13), no validation in an independent sample was possible. The mediation model used concurrent measures and cannot establish that literacy preceded disclosure confidence or that either caused adoption intention. Competing specifications with the ordering reversed fitted the data equally well (Table 10), so the indirect estimate should be described as an indirect statistical association rather than as mediation in the causal sense; longitudinal measurement of literacy, confidence, and adoption is required for meaningful mediation inference. It was also sensitive to modest unmeasured mediator–outcome confounding and was somewhat underpowered for an indirect effect of the observed size.

Future studies should recruit multicenter national and European samples, test measurement invariance across professional roles, and follow participants longitudinally as AI systems are implemented. Such designs would also permit external validation of the implementation profiles against prespecified criteria in independent samples. Training and governance interventions could be evaluated using prespecified outcomes such as objective literacy, calibration of trust, quality of simulated disclosure, completeness of incident reports, override behavior, and real-world adoption. Linking survey measures to de-identified AI-event data would help determine whether the identified attitudes predict safer practice. Given the rapid evolution of generative and multimodal systems, future instruments should also assess model-specific risks, human oversight, and post-deployment monitoring, distinguish explicitly between predictive, generative, and agentic or multi-agent systems, because liability perceptions may not generalize equally across these technologies, and capture the conditions under which professionals would decline to use, override, or escalate an AI output, in line with updated international guidance (Challen et al., 2019; Char et al., 2018; Wiens et al., 2019; World Health Organization, 2025).

## 5. Conclusions

In this exploratory, single-center study of a Romanian healthcare network, readiness to adopt clinical AI was associated with objective liability literacy, confidence in disclosure and reporting, trust in AI, lower defensive attitudes, and previous AI-error training. Associations with liability literacy, trust, and disclosure confidence persisted in penalized sensitivity analyses, whereas those with defensive stance and previous training did not. The principal associations were attenuated but not removed by adjustment for professional role and were similar among current users and non-users of AI; the indirect association through disclosure confidence should be regarded as a statistical decomposition rather than as a mediating pathway. The marked disparity between familiarity with the GDPR and awareness of AI-specific regulation suggests that privacy education alone may be insufficient for accountable implementation. The appropriate goal is calibrated rather than maximal adoption, in which willingness to use AI is conditional on validation, oversight, and escalation routes. The findings provide a preliminary rationale, which requires confirmation, for pairing role-specific education with explicit human-oversight responsibilities, accessible reporting and disclosure pathways, auditability, and non-punitive multidisciplinary review. The exploratory implementation profiles may help target support, but they describe this sample only and require validation against external criteria in independent settings. Because the sample was small, the analyses were exploratory and unadjusted for multiplicity, and several estimates were imprecise, these results should be treated as hypothesis-generating and should not yet be used to guide institutional policy or the assessment of individual professionals. Multicenter longitudinal and intervention studies are needed to determine whether improving literacy and governance produces safer observed AI use and more effective responses to AI-related errors.

## Figures and Tables

**Figure 1:**
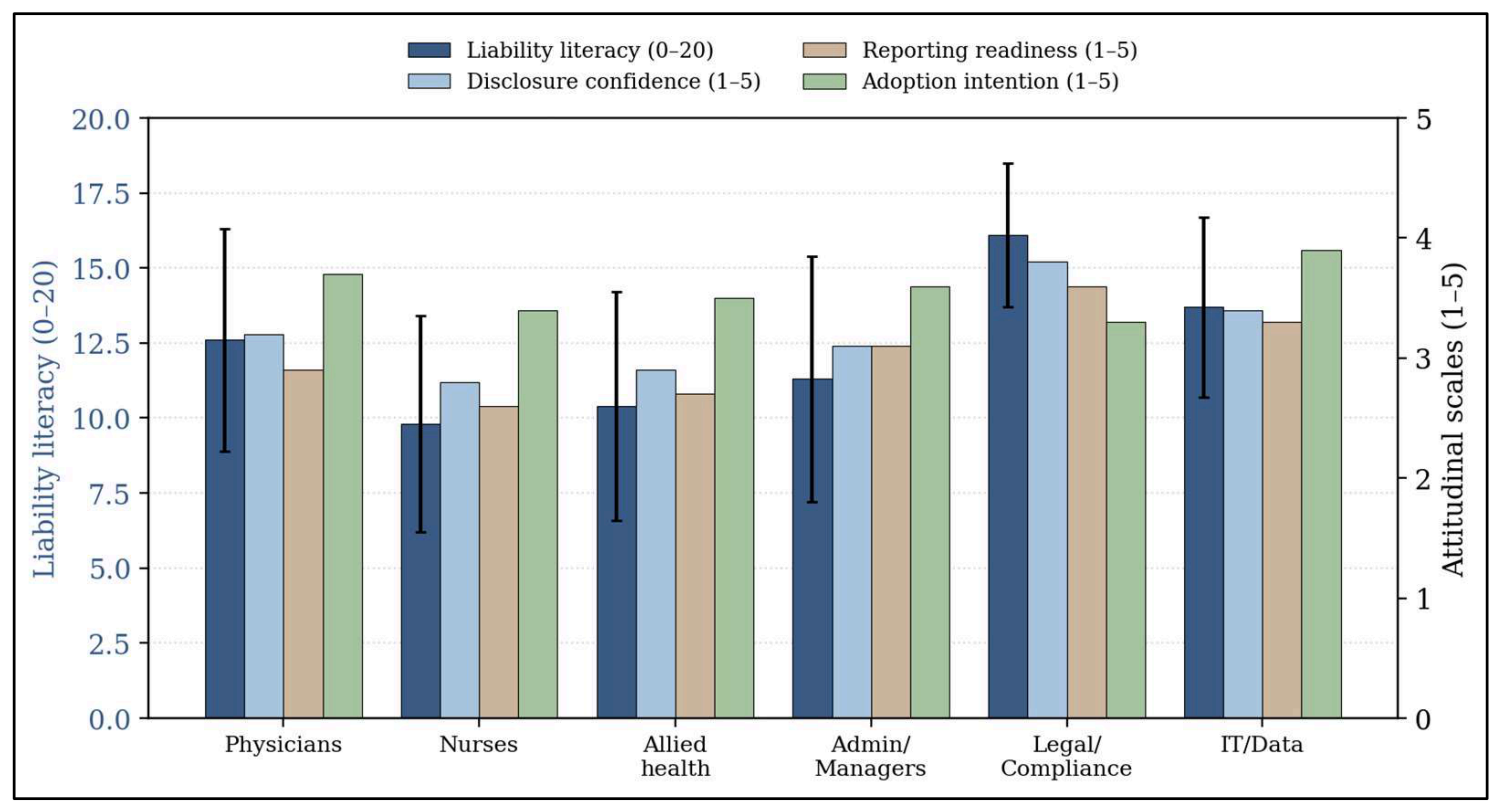
Professional-role means for liability literacy, disclosure confidence, reporting readiness, and adoption intention. Error bars indicate standard deviations. Between-role differences were significant for liability literacy (*p* < 0.001), disclosure confidence (*p* = 0.013), and reporting readiness (*p* = 0.003), but not for adoption intention (*p* = 0.302).

**Figure 2:**
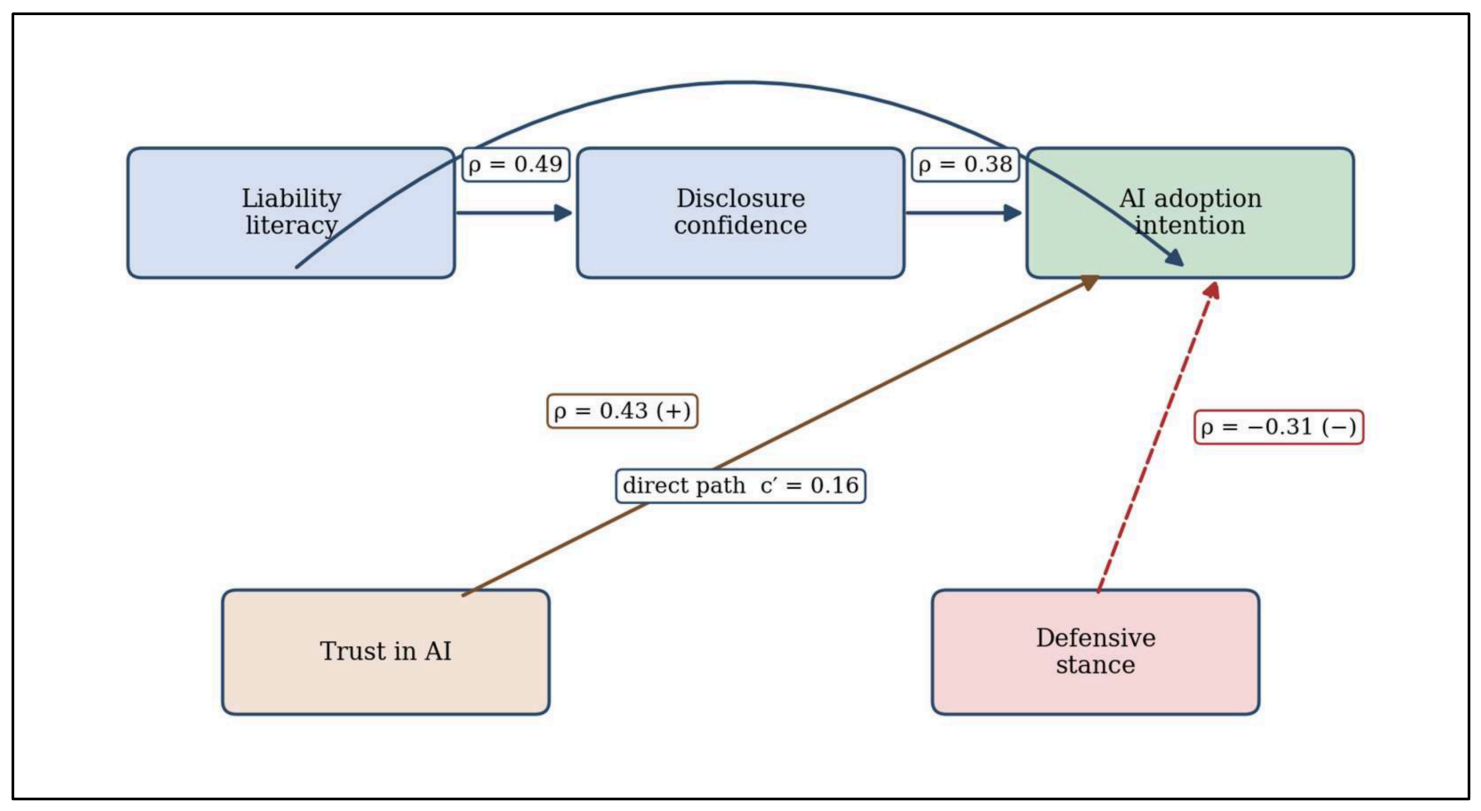
Conceptual representation of the observed relationships among liability literacy, disclosure confidence, trust, defensive stance, and AI adoption intention. Values marked ρ are Spearman correlations; c′ is the adjusted direct association between literacy and adoption intention in the mediation model.

**Figure 3:**
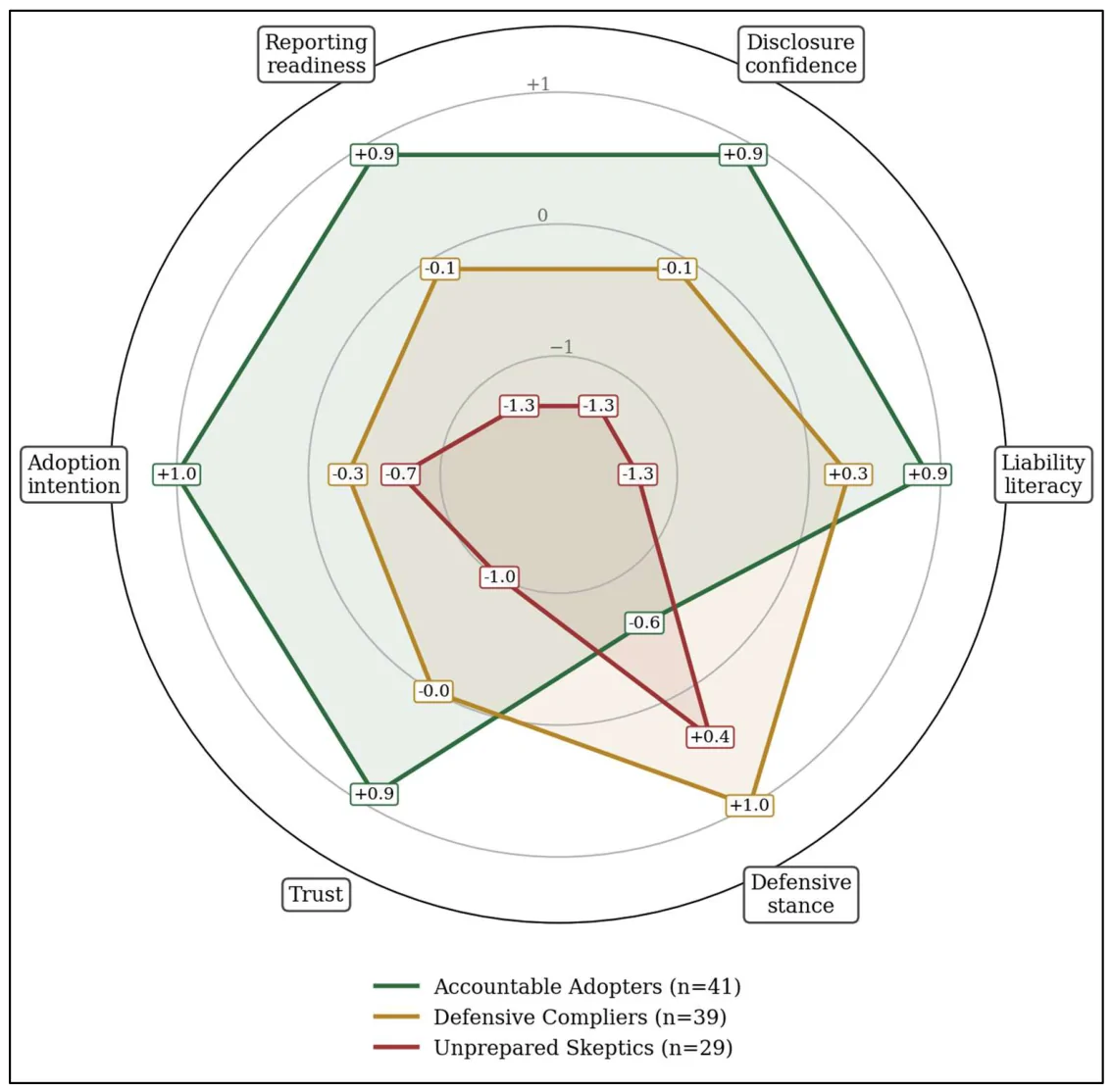
Standardized construct patterns for the three k-means implementation profiles. Lines show profile means expressed as z-scores for the measures included in clustering; zero represents the full-sample mean.

**Table 1:** Demographic and professional characteristics of the study participants (*N* = 109).

Characteristic	Value
Age, years	39.4 ± 10.1
Female sex	67 (61.5%)
Sector: Public hospital	56 (51.4%)
Sector: Private clinic	31 (28.4%)
Sector: Academic/University	22 (20.2%)
Profession: Physicians	33 (30.3%)
Profession: Nurses	27 (24.8%)
Profession: Allied health	12 (11.0%)
Profession: Administrators/Managers	16 (14.7%)
Profession: Legal/Compliance	11 (10.1%)
Profession: IT/Data	10 (9.2%)
Prior AI-error training (any)	39 (35.8%)
Witnessed an AI error/near-miss	43 (39.4%)
Reported AI use in workflow (any)	47 (43.1%)

**Table 2:** Liability literacy, disclosure confidence, reporting readiness, and adoption intention by professional role (mean ± SD).

Role	*n*	Liability Literacy (0–20)	Disclosure Confidence (1–5)	Reporting Readiness (1–5)	Adoption Intention (1–5)
Physicians	33	12.6 ± 3.7	3.2 ± 0.8	2.9 ± 0.8	3.7 ± 0.7
Nurses	27	9.8 ± 3.6	2.8 ± 0.8	2.6 ± 0.7	3.4 ± 0.8
Allied health	12	10.4 ± 3.8	2.9 ± 0.9	2.7 ± 0.8	3.5 ± 0.7
Administrators/Managers	16	11.3 ± 4.1	3.1 ± 0.8	3.1 ± 0.7	3.6 ± 0.8
Legal/Compliance	11	16.1 ± 2.4	3.8 ± 0.6	3.6 ± 0.6	3.3 ± 0.7
IT/Data	10	13.7 ± 3.0	3.4 ± 0.7	3.3 ± 0.7	3.9 ± 0.6
Overall	109	11.6 ± 4.2	3.1 ± 0.9	2.9 ± 0.8	3.6 ± 0.8

Note: SD, standard deviation. One-way ANOVA *p*-values: liability literacy, *p* < 0.001; disclosure confidence, *p* = 0.013; reporting readiness, *p* = 0.003; adoption intention, *p* = 0.302.

**Table 3:** Self-reported familiarity with the legal frameworks most relevant to clinical AI (*N* = 109).

Framework	Familiar (*n*, %)
General Data Protection Regulation (GDPR)	74 (67.9%)
Medical Device Regulation (MDR)	49 (45.0%)
Romanian Law no. 190/2018 (GDPR implementing measures)	33 (30.3%)
EU Artificial Intelligence Act	27 (24.8%)
2022 AI Liability Directive proposal	22 (20.2%)

Note: Familiarity reflects self-report and is not equivalent to objective liability literacy. The 2022 AI Liability Directive proposal was formally withdrawn in 2025 (European Commission, 2025).

**Table 4:** Comparison of outcomes between participants with and without prior experience of an AI error or near-miss.

Outcome	Witnessed AI Error/Near-Miss (*n* = 43)	No Prior AI Error/Near-Miss (*n* = 66)	*p*-Value
Liability literacy (0–20)	13.1 ± 3.6	10.7 ± 4.1	0.002
Disclosure confidence (1–5)	3.4 ± 0.8	2.9 ± 0.8	0.002
Defensive stance (1–5)	3.6 ± 0.7	3.2 ± 0.8	0.007
Reporting readiness (1–5)	3.2 ± 0.7	2.8 ± 0.8	0.007
Adoption intention (1–5)	3.5 ± 0.8	3.6 ± 0.7	0.505

Note: Independent-samples Welch *t*-tests. Higher defensive-stance scores indicate greater risk-averse practice change.

**Table 5:** Spearman rank correlations among the key study measures (*N* = 109).

Pair	ρ	*p*-Value
Literacy vs. Disclosure confidence	0.49	<0.001
Disclosure confidence vs. Adoption intention	0.38	<0.001
Trust vs. Adoption intention	0.43	<0.001
Defensive stance vs. Adoption intention	−0.31	0.001
Literacy vs. Reporting readiness	0.34	<0.001
Defensive stance vs. Disclosure confidence	−0.27	0.005

Note: ρ, Spearman rank correlation. Confidence intervals were obtained by the Fisher z transformation: literacy–disclosure confidence, 0.33 to 0.62; disclosure confidence–adoption intention, 0.21 to 0.53; trust–adoption intention, 0.26 to 0.57; defensive stance–adoption intention, −0.47 to −0.13; literacy–reporting readiness, 0.16 to 0.50; defensive stance–disclosure confidence, −0.44 to −0.09. *p*-values are unadjusted for multiple comparisons.

**Table 6:** Role-adjusted sensitivity analysis of the bivariate correlations (*N* = 109).

Pair	Pooled ρ (95% CI)	Partial ρ, Role-Adjusted (95% CI)	Within-Role ρ, Random Intercept (95% CI)
Literacy vs. Disclosure confidence	0.49 (0.33, 0.62)	0.41 (0.24, 0.56)	0.40 (0.22, 0.55)
Disclosure confidence vs. Adoption intention	0.38 (0.21, 0.53)	0.36 (0.18, 0.52)	0.35 (0.17, 0.51)
Trust vs. Adoption intention	0.43 (0.26, 0.57)	0.41 (0.24, 0.56)	0.40 (0.22, 0.55)
Defensive stance vs. Adoption intention	−0.31 (−0.47, −0.13)	−0.29 (−0.46, −0.10)	−0.28 (−0.45, −0.09)
Literacy vs. Reporting readiness	0.34 (0.16, 0.50)	0.27 (0.08, 0.44)	0.26 (0.07, 0.43)
Defensive stance vs. Disclosure confidence	−0.27 (−0.44, −0.09)	−0.24 (−0.41, −0.05)	−0.23 (−0.40, −0.04)

Note: ρ, Spearman rank correlation; CI, confidence interval. Partial correlations were computed between residuals after regression of each measure on five professional-role indicator variables; within-role correlations were obtained from a bivariate random-intercept model with professional role as the grouping factor. Confidence intervals used the Fisher z transformation, with degrees of freedom reduced for the role indicators.

**Table 7:** Multivariable logistic regression model of factors associated with high adoption intention (score ≥ 4).

Predictor	aOR (95% CI)	*p*-Value
Liability literacy (per +1 SD)	1.78 (1.16–2.73)	0.008
Trust (per +1 SD)	1.69 (1.10–2.59)	0.016
Disclosure confidence (per +1 SD)	1.61 (1.04–2.49)	0.033
Defensive stance (per +1 SD)	0.66 (0.44–0.98)	0.042
Prior AI-error training (yes vs. no)	2.04 (1.01–4.12)	0.047
Age (per 10 years)	0.94 (0.69–1.28)	0.695
Female sex (vs. male)	1.11 (0.55–2.24)	0.771
Public sector (vs. private/academic)	0.85 (0.42–1.72)	0.651

Note: aOR, adjusted odds ratio; CI, confidence interval; SD, standard deviation. The model was adjusted for age, sex, sector, and professional role; high adoption intention was reported by 53/109 participants (48.6%).

**Table 8:** Characteristics of the three implementation profiles derived by k-means clustering (mean ± SD).

Variable	AccountableAdopters (*n* = 41)	DefensiveCompliers (*n* = 39)	UnpreparedSkeptics (*n* = 29)	*p*-Value
Liability literacy (0–20)	13.4 ± 3.4	11.8 ± 3.7	8.1 ± 3.6	<0.001
Disclosure confidence (1–5)	3.6 ± 0.6	3.0 ± 0.7	2.3 ± 0.7	<0.001
Reporting readiness (1–5)	3.4 ± 0.6	2.9 ± 0.7	2.2 ± 0.6	<0.001
Adoption intention (1–5)	4.1 ± 0.5	3.3 ± 0.6	3.1 ± 0.7	<0.001
Trust in AI (1–5)	3.7 ± 0.5	3.2 ± 0.6	2.8 ± 0.6	<0.001
Defensive stance (1–5)	3.1 ± 0.6	4.0 ± 0.5	3.7 ± 0.7	<0.001

Note: Profiles were derived from k-means clustering on standardized measures; *p*-values are from one-way ANOVA and are shown for completeness only, because these six measures were the inputs to the clustering algorithm, between-profile differences on them are expected by construction and should be read descriptively rather than as evidence of profile validity. Internal validation of this solution and an external comparison of the profiles on variables not used in clustering are reported in Section 3.10.

**Table 9:** Covariate-adjusted mediation model of the pathway from liability literacy to adoption intention through disclosure confidence.

Path	Estimate (β)	SE	*p*-Value	95% CI
a: Literacy → Confidence	0.43	0.08	<0.001	—
b: Confidence → Adoption	0.27	0.09	0.003	—
c′: Literacy → Adoption (direct)	0.16	0.07	0.024	—
c: Literacy → Adoption (total)	0.28	0.08	<0.001	—
Indirect (a × b), bootstrap (B = 5000)	0.12	—	—	[0.04, 0.22]

Note: Outcome = adoption intention (1–5). Covariates: age, sex, sector, professional role, and prior AI-error training. —, not applicable.

**Table 10:** Competing mediation specifications estimated with the same covariates (*N* = 109).

Specification	a Path, β (SE)	b Path, β (SE)	Direct Path c′, β (SE)	Indirect Estimate (95% Bootstrap CI)
A (primary): Literacy → Confidence → Adoption	0.43 (0.08)	0.27 (0.09)	0.16 (0.07)	0.12 [0.04, 0.22]
B (reversed): Confidence → Literacy → Adoption	0.41 (0.08)	0.16 (0.07)	0.27 (0.09)	0.07 [0.01, 0.15]
C (alternative): Literacy → Adoption → Confidence	0.28 (0.08)	0.24 (0.09)	0.36 (0.08)	0.07 [0.01, 0.14]

Note: β, standardized coefficient; SE, standard error; CI, confidence interval. Covariates: age, sex, sector, professional role, and prior AI-error training; indirect estimates from 5000 bias-corrected bootstrap resamples. The three specifications are statistically equivalent under cross-sectional data and are shown to illustrate the dependence of the interpretation on assumed directionality, not to adjudicate among them.

**Table 11:** Sensitivity analyses for the multivariable logistic model of high adoption intention (*N* = 109; 53 events).

Predictor/Model Characteristic	Fully Adjusted Model, aOR (95% CI)	Firth Penalized Model, aOR (95% CI)	Parsimonious Model, aOR (95% CI)
Liability literacy (per +1 SD)	1.78 (1.16–2.73)	1.69 (1.12–2.58)	1.83 (1.22–2.75)
Trust (per +1 SD)	1.69 (1.10–2.59)	1.62 (1.07–2.45)	1.72 (1.15–2.58)
Disclosure confidence (per +1 SD)	1.61 (1.04–2.49)	1.54 (1.02–2.34)	1.58 (1.06–2.36)
Defensive stance (per +1 SD)	0.66 (0.44–0.98)	0.70 (0.48–1.02)	0.68 (0.47–0.98)
Prior AI-error training (yes vs. no)	2.04 (1.01–4.12)	1.88 (0.95–3.72)	1.97 (1.00–3.88)
Parameters estimated	13	13	5
Events per estimated parameter	4.1	4.1	10.6
Apparent c-statistic (95% CI)	0.79 (0.70–0.87)	0.78 (0.69–0.86)	0.76 (0.67–0.85)
Optimism-corrected c-statistic	0.72	0.72	0.71
Bootstrap calibration slope	0.78	0.86	0.83

Note: aOR, adjusted odds ratio; CI, confidence interval; SD, standard deviation. The fully adjusted and Firth models included age, sex, sector, and professional role; the parsimonious model included only the five key predictors. Optimism-corrected statistics were derived from 1000 bootstrap replications. Hosmer–Lemeshow test for the fully adjusted model: χ^2^ = 8.12, df = 8, *p* = 0.42; maximum variance inflation factor = 1.74.

**Table 12:** Internal validation of the three k-means implementation profiles (*N* = 109).

Validation Procedure	Result	Interpretation
Average silhouette width (k = 2/3/4/5)	0.34/0.41/0.29/0.24	k = 3 optimal; separation only moderate
Gap statistic (500 reference datasets)	Maximum at k = 3	Supports three-cluster solution
Calinski–Harabasz index (k = 2/3/4/5)	51.4/62.8/54.3/47.9	Supports three-cluster solution
Convergence across 1000 random starts	Identical partition in 96.4%	Not dependent on initialization
Bootstrap Jaccard, Accountable Adopters	0.82 (dissolved in 2.1%)	Stable
Bootstrap Jaccard, Defensive Compliers	0.71 (dissolved in 8.4%)	Pattern present but uncertain
Bootstrap Jaccard, Unprepared Skeptics	0.76 (dissolved in 5.3%)	Stable (near lower boundary of band)
Agreement with Ward hierarchical clustering	ARI = 0.68; 92/109 (84.4%) identical	Moderate-to-substantial agreement
Agreement with partitioning around medoids	ARI = 0.73; 95/109 (87.2%) identical	Substantial agreement
Leave-one-variable-out reassignment	ARI range 0.61–0.88	No single measure drives the solution
Observations per clustering variable (smallest profile)	4.8	Below recommended thresholds

Note: ARI, adjusted Rand index. Bootstrap resampling used 1000 replications; a mean Jaccard similarity ≥ 0.85 indicates a highly stable cluster, 0.75–0.85 a stable cluster, 0.60–0.75 a pattern that is present but uncertain, and <0.60 an unstable cluster. Dissolution denotes a replication in which the maximum Jaccard similarity for the cluster fell below 0.50.

**Table 13:** Comparison of the three implementation profiles on variables that were not used in clustering.

Variable	Accountable Adopters (*n* = 41)	Defensive Compliers (*n* = 39)	Unprepared Skeptics (*n* = 29)	*p*-Value
Age, years (mean ± SD)	38.6 ± 9.7	41.2 ± 10.4	38.1 ± 10.2	0.375
Female sex, *n* (%)	25 (61.0%)	25 (64.1%)	17 (58.6%)	0.897
Public-hospital sector, *n* (%)	20 (48.8%)	21 (53.8%)	15 (51.7%)	0.902
Clinical role (physician, nurse, allied health), *n* (%)	26 (63.4%)	26 (66.7%)	20 (69.0%)	0.885
Prior AI-error training, *n* (%)	21 (51.2%)	13 (33.3%)	5 (17.2%)	0.013
Witnessed an AI error/near-miss, *n* (%)	20 (48.8%)	18 (46.2%)	5 (17.2%)	0.016
Current AI use in workflow, *n* (%)	25 (61.0%)	15 (38.5%)	7 (24.1%)	0.007
Familiar with the EU AI Act, *n* (%)	16 (39.0%)	8 (20.5%)	3 (10.3%)	0.018

Note: SD, standard deviation. None of these variables entered the k-means algorithm. *p*-values are from one-way ANOVA (age) or chi-square tests (categorical variables) and are unadjusted for multiple comparisons; Cramér’s V for the four significant contrasts ranged from 0.27 to 0.30.

**Table 14:** Sensitivity analysis distinguishing current users of AI in the workflow from non-users.

Measure	Current AI Users (*n* = 47)	Non-Users (*n* = 62)	*p*-Value
Liability literacy (0–20)	12.8 ± 4.0	10.7 ± 4.1	0.009
Disclosure confidence (1–5)	3.3 ± 0.8	3.0 ± 0.9	0.069
Reporting readiness (1–5)	3.1 ± 0.7	2.8 ± 0.8	0.040
Trust in AI (1–5)	3.5 ± 0.6	3.1 ± 0.7	0.002
Defensive stance (1–5)	3.3 ± 0.8	3.4 ± 0.8	0.520
Adoption intention (1–5)	3.9 ± 0.6	3.3 ± 0.8	<0.001
High adoption intention (score ≥ 4), *n* (%)	30 (63.8%)	23 (37.1%)	0.006
ρ, literacy vs. adoption intention (95% CI)	0.31 (0.03, 0.55)	0.33 (0.09, 0.54)	—
ρ, disclosure confidence vs. adoption intention (95% CI)	0.35 (0.07, 0.58)	0.39 (0.16, 0.58)	—
ρ, trust vs. adoption intention (95% CI)	0.40 (0.13, 0.62)	0.44 (0.21, 0.62)	—

Note: Values are mean ± SD unless stated otherwise. *p*-values are from Welch *t*-tests or a chi-square test; ρ, Spearman rank correlation within stratum, with Fisher z confidence intervals. Adding current use as a covariate to the fully adjusted logistic model gave aORs of 1.72 (1.10–2.69) for liability literacy, 1.63 (1.05–2.53) for trust, 1.57 (1.00–2.46) for disclosure confidence, and 2.61 (1.12–6.08) for current use itself; use × predictor interaction terms were non-significant (all *p* ≥ 0.41). —, not applicable.

## Data Availability

The data presented in this study are available on request from the corresponding author. The analysis code for the sensitivity, internal-validation, and cluster-stability procedures reported in Section 3.5 and Section 3.8, Section 3.9, Section 3.10 and Section 3.11 is likewise available from the corresponding author on request.

## Appendix A

Table A1 maps each item of the 20-item AI Error Liability Literacy Index to its content domain and primary legal source. Item content is summarized by topic; the full instrument, with scoring key, is available from the corresponding author on request.

**Table A1:** Mapping of the 20 items of the AI Error Liability Literacy Index to content domains and primary legal sources.

Item	Topic Assessed (Paraphrased)	Content Domain	Primary Legal Source
1	Risk classification of AI that is, or is a safety component of, a medical device	AI Act obligations	AI Act, Art. 6 and Annex I
2	Human-oversight measures required for high-risk systems	AI Act obligations	AI Act, Art. 14
3	Deployer duties to assign oversight, monitor operation, and follow instructions for use	AI Act obligations	AI Act, Art. 26
4	Automatic logging and record-keeping that enable traceability	AI Act obligations	AI Act, Art. 12
5	Reporting of serious incidents and the associated timelines	AI Act obligations	AI Act, Art. 73
6	Transparency to deployers and AI-literacy expectations for staff	AI Act obligations	AI Act, Art. 4 and Art. 13
7	Software as a medical device and rule-based classification	Medical device regulation	MDR, Art. 2 and Annex VIII, Rule 11
8	Manufacturer vigilance and post-market surveillance duties	Medical device regulation	MDR, Art. 83 and Art. 87
9	Use outside the intended purpose or CE-marked configuration	Medical device regulation	MDR, Art. 5 and Art. 20
10	Software as a “product” and the concept of defectiveness	Product and non-contractual liability	PLD, Art. 4 and Art. 7
11	Economic operators who may be held liable for a defective AI product	Product and non-contractual liability	PLD, Art. 8
12	Disclosure of evidence and presumptions of defectiveness or causation in claims	Product and non-contractual liability	PLD, Art. 9–10; AILD proposal, Art. 3–4
13	Continuing role of national fault-based rules for professional negligence	Product and non-contractual liability	AILD proposal, Art. 1 (scope); national civil-liability rules
14	Health data as a special category and lawful bases for processing	Data protection	GDPR, Art. 9; Law no. 190/2018, Art. 3
15	Rights concerning solely automated decisions and human intervention	Data protection	GDPR, Art. 22
16	Data-protection impact assessment before high-risk processing	Data protection	GDPR, Art. 35
17	Personal-data breach notification to the authority and to patients	Data protection	GDPR, Art. 33–34; Law no. 190/2018
18	Which parties must be notified after an AI-related adverse event and through which channel?	Cross-cutting disclosure, documentation, and escalation	AI Act, Art. 26(5) and Art. 73; MDR, Art. 87
19	Documentation needed to reconstruct an event (inputs, system version, output, override, log retention)	Cross-cutting disclosure, documentation, and escalation	AI Act, Art. 12 and Art. 26(6)
20	When to preserve evidence and seek legal or compliance consultation	Cross-cutting disclosure, documentation, and escalation	PLD, Art. 9; AILD proposal, Art. 3

Note: AI Act, Regulation (EU) 2024/1689; MDR, Medical Device Regulation (EU) 2017/745; PLD, Product Liability Directive (EU) 2024/2853; GDPR, General Data Protection Regulation (EU) 2016/679; AILD proposal, proposed AI Liability Directive, COM (2022) 496 final (withdrawn in 2025). Items 12, 13, and 20 were scored on the basis of the general principles of the proposal and of the recast Product Liability Directive rather than on provisions of the withdrawn text. Topics are paraphrased; full item wording is available from the corresponding author.
